# WW, PH and C-Terminal Domains Cooperate to Direct the Subcellular Localizations of PLEKHA5, PLEKHA6 and PLEKHA7

**DOI:** 10.3389/fcell.2021.729444

**Published:** 2021-09-09

**Authors:** Sophie Sluysmans, Isabelle Méan, Lionel Jond, Sandra Citi

**Affiliations:** Department of Cell Biology, Faculty of Sciences, University of Geneva, Geneva, Switzerland

**Keywords:** pleckstrin homology domain-containing family A, WW domain, PDZD11, PLEKHA5, PLEKHA6, PLEKHA7

## Abstract

PLEKHA5, PLEKHA6, and PLEKHA7 (WW-PLEKHAs) are members of the PLEKHA family of proteins that interact with PDZD11 through their tandem WW domains. WW-PLEKHAs contribute to the trafficking and retention of transmembrane proteins, including nectins, Tspan33, and the copper pump ATP7A, at cell-cell junctions and lateral membranes. However, the structural basis for the distinct subcellular localizations of PLEKHA5, PLEKHA6, and PLEKHA7 is not clear. Here we expressed mutant and chimeric proteins of WW-PLEKHAs in cultured cells to clarify the role of their structural domains in their localization. We found that the WW-mediated interaction between PLEKHA5 and PDZD11 is required for their respective association with cytoplasmic microtubules. The PH domain of PLEKHA5 is required for its localization along the lateral plasma membrane and promotes the lateral localization of PLEKHA7 in a chimeric molecule. Although the PH domain of PLEKHA7 is not required for its localization at the adherens junctions (AJ), it promotes a AJ localization of chimeric proteins. The C-terminal region of PLEKHA6 and PLEKHA7 and the coiled-coil region of PLEKHA7 promote their localization at AJ of epithelial cells. These observations indicate that the localizations of WW-PLEKHAs at specific subcellular sites, where they recruit PDZD11, are the result of multiple cooperative protein-lipid and protein-protein interactions and provide a rational basis for the identification of additional proteins involved in trafficking and sorting of WW-PLEKHAs.

## Introduction

**FIGURE 1:**
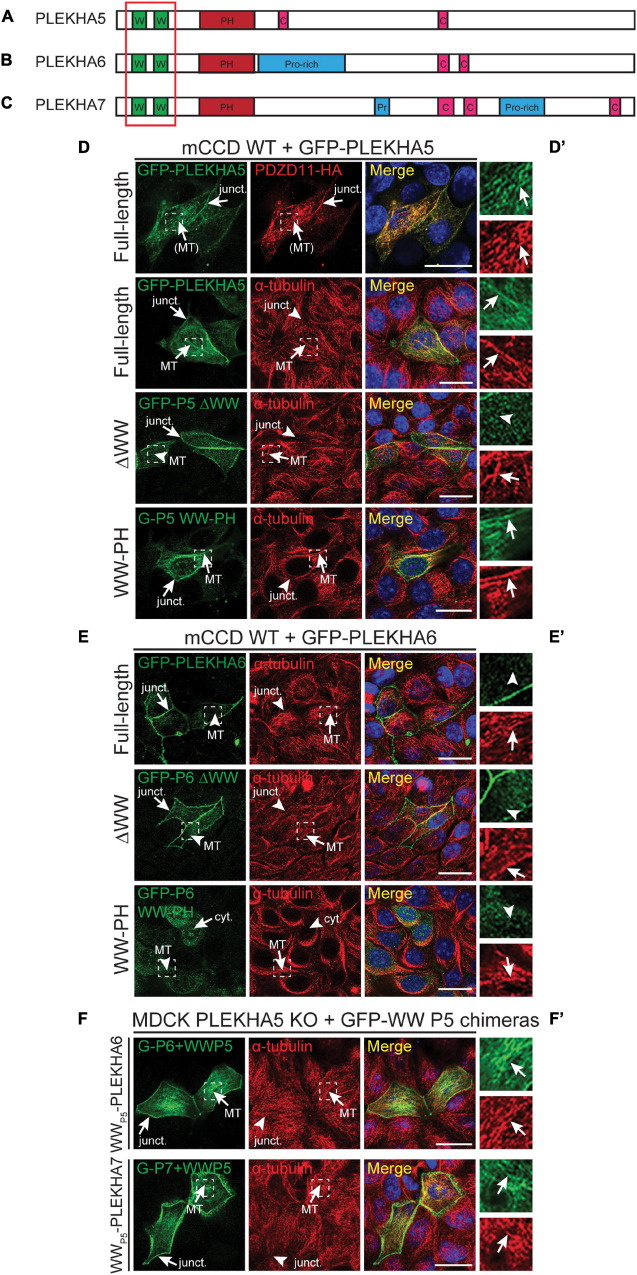
The tandem WW domains of PLEKHA5 stabilize its localization along microtubules. **(A–C)** Scheme of the structural organization of PLEKHA5 **(A)**, PLEKHA6 **(B)**, and PLEKHA7 **(C)** showing the domains: WW (Trp-Trp) (W) in green, PH (pleckstrin homology) in red, Proline-rich (Pro-rich or Pr) in blue, coiled-coil (C) in pink. Red box indicates the structural domains analyzed. **(D,E)** IF analysis of the localization of GFP-tagged PLEKHA5 **(D)** and PLEKHA6 **(E)** constructs, either full-length, or a deletion lacking the WW domains (ΔWW), or the isolated N-terminal WW-PH region (WW-PH) after transfection in WT mCCD cells. **(F)** IF analysis of the localization of GFP-tagged chimeras of either PLEKHA6 or PLEKHA7, containing the WW domains of PLEKHA5 (P5), expressed in PLEKHA5 KO MDCK cells. α-tubulin is used as a marker for microtubules. In panels **(D–F)**, squares correspond to enlarged areas in panels **(D′–F′)**. Arrows show labeling, arrowheads indicate low/undetectable labeling. Junctional (junct.), fibrillar microtubules-like [(MT)], microtubules (MT) and cytoplasmic (cyt.) localizations are indicated. Scale bar = 20 μm.

The PLEKHA (Pleckstrin Homology domain containing family A) family of proteins is characterized by the presence of a pleckstrin homology (PH) domain and comprises PLEKHA1 and PLEKHA2 (TAPP1 and TAPP2) (Kimber et al., 2002; Marshall et al., 2002), PLEKHA3 and PLEKHA8 (FAPP1 and FAPP2) (Godi et al., 2004), PLEKHA4 (PEPP1) (Dowler et al., 2000; Shami Shah et al., 2019), PLEKHA5 and PLEKHA6 (PEPP2 and PEPP3) (Dowler et al., 2000; Zou and Cox, 2013; Sluysmans et al., 2021), and PLEKHA7 (Meng et al., 2008; Pulimeno et al., 2010). We denoted as WW-PLEKHAs a subset of the PLEKHA family of proteins, i.e., PLEKHA5, PLEKHA6, and PLEKHA7, which include isoforms that also contain Trp-Trp (WW) domains, in addition to the characteristic PH domain (Sluysmans et al., 2021). All three WW-PLEKHA proteins also contain proline-rich and coiled-coil domains of unknown function (Figures 1A–C).

WW-PLEKHAs together with PDZD11 control the targeting of the Menkes copper pump ATP7A to the cell periphery under elevated copper conditions (Sluysmans et al., 2021). Furthermore, PLEKHA7 together with PDZD11 controls the localization of nectins, and of the Tspan33-ADAM10 complex at adherens junctions (AJ) (Guerrera et al., 2016; Shah et al., 2018). PLEKHA5 colocalizes with microtubules in the cytoplasm of epithelial, endothelial and myeloblastic-derived cells and when exogenously expressed recruits PDZD11 to this localization (Sluysmans et al., 2021). PLEKHA6 is localized at AJ and along lateral membranes of polarized epithelial cells, and PLEKHA7 exclusively at AJ of epithelial and non-epithelial cells (Pulimeno et al., 2010; Shah et al., 2018; Sluysmans et al., 2021). However, the structural basis for the specific subcellular localizations of WW-PLEKHAs is unclear. Here we explore the role of PH, WW, and coiled-coil domains of each of the three WW-PLEKHAs in determining their subcellular localizations, by expressing mutant and chimeric proteins in epithelial cells. The results indicate specific roles for WW, PH and coiled-coil domains in orchestrating the subcellular localizations of distinct WW-PLEKHAs.

## Materials and Methods

Key resources used in this manuscript are listed in Table 1.

**TABLE 1:** Key resources.

**Reagent or resource**	**Source**	**Identifier**
**Antibodies**		
Rabbit polyclonal anti-PDZD11	Citi Laboratory (Guerrera et al., 2016)	Rb29958
Guinea pig polyclonal anti-PLEKHA7	Citi Laboratory (Guerrera et al., 2016)	GP2737
Mouse monoclonal anti-β-tubulin	Thermo Fisher Scientific	Cat# 32-2600, RRID: AB_2533072
Mouse monoclonal anti-α-tubulin	Thermo Fisher Scientific	Cat# 32-2500, RRID: AB_2533071
Guinea pig monoclonal anti-α-tubulin	Geneva Antibody Facility (Guerreiro and Meraldi (2019). Antibody Reports)	AA345 scFv-F2C
Rabbit polyclonal anti-GFP	Thermo Fisher Scientific	Cat# A-11122, RRID: AB_221569
Mouse monoclonal anti-HA	Thermo Fisher Scientific	Cat# 32-6700, RRID: AB_2533092
Mouse monoclonal anti-E-cadherin	BD Biosciences	Cat# 610181, RRID: AB_397580
Rat monoclonal anti-ZO-1	Goodenough Laboratory (Harvard Medical School)	R40.76 RRID: AB_2205518
Rabbit polyclonal anti-GST	Thermo Fisher Scientific	Cat# 71-7500 RRID: AB_2533994
Alexa Fluor 488-AffiniPure Donkey anti-Rabbit IgG	Jackson ImmunoResearch	Cat# 711-545-152, RRID: AB_2313584
Cy3-AffiniPure Donkey anti-Mouse IgG	Jackson ImmunoResearch	Cat# 715-165-151, RRID: AB_2315777
Alexa Fluor 647-AffiniPure Donkey anti-Guinea Pig IgG	Jackson ImmunoResearch	Cat# 706-605-148, RRID: AB_2340476
Cy5-AffiniPure Donkey anti-Rat IgG	Jackson ImmunoResearch	Cat# 712-175-153, RRID: AB_2340672
Anti-Mouse IgG (H + L), HRP conjugate	Promega	Cat# W4021, RRID: AB_430834
Anti-Rabbit IgG (H + L), HRP conjugate	Promega	Cat# W4011, RRID: AB_430833
**Bacterial Strains**		
BL21-DE3 Competent cells	NEB	Cat# C2530H
DH5-α Competent cells	Thermo Fisher	Cat# 18265017
**Chemicals and Recombinant Proteins**		
Poly-L-Lysine solution	Sigma-Aldrich	Cat# P4707
MG132	Sigma-Aldrich	Cat# C2211
GST-human PLEKHA5-PH (120-270)	Citi Laboratory (This paper)	S2090
GST-human PLEKHA6-PH (120-285)	Citi Laboratory (This paper)	S2095
GST-human PLEKHA7-PH (120-300)	Citi Laboratory (Guerrera et al., 2016)	S1795
**Critical Commercial Assays**		
jetOPTIMUS DNA Transfection Reagent	Polyplus	Cat# 117-15
3.5 kDa-MWCO dialysis membrane (Spectra/Por3 3,500 MWCO 18mm)	Spectrum Laboratories	Cat# 132720
PIP Strips	Echelon Biosciences	Cat# P-60001
Pierce Glutathione Magnetic Agarose Beads	Thermo Fisher Scientific	Cat# 78602
Matrigel	BD Biosciences	Cat# 354230
Protease inhibitor cocktail	Thermo Fisher Scientific	Cat# A32965
**Experimental Models: Cell Lines**		
Mouse cortical collecting duct cell line, mCCD WT N64-Tet-ON	Feraille Laboratory, Unige	N/A
Mouse cortical collecting duct cell line, mCCD PLEKHA7-KO N64-Tet-ON	Citi Laboratory (Guerrera et al., 2016; Shah et al., 2018)	N/A
Mouse cortical collecting duct cell line, mCCD PDZD11-KO N64-Tet-ON	Citi Laboratory (Guerrera et al., 2016)	N/A
Mouse cortical collecting duct cell line, mCCD PLEKHA6-KO N64-Tet-ON	Citi Laboratory (Sluysmans et al., 2021)	N/A
Mouse cortical collecting duct cell line, mCCD PLEKHA6/7-KO N64-Tet-ON	Citi Laboratory (Sluysmans et al., 2021)	N/A
Canine kidney proximal tubule cell line, MDCK-II WT Tet-OFF	Fanning Laboratory, U. North Carolina	N/A
Canine kidney proximal tubule cell line, MDCK-II PLEKHA5-KO Tet-OFF	Citi Laboratory (Sluysmans et al., 2021)	N/A
Canine kidney proximal tubule cell line, MDCK-II PLEKHA6-KO Tet-OFF	Citi Laboratory (Sluysmans et al., 2021)	N/A
Human haploid cell lines, Hap1 WT, PLEKHA7-KO	Amieva Laboratory, Standford (Popov et al., 2015)	N/A
Human haploid cell line, Hap1 PDZD11-KO	Citi Laboratory (Shah et al., 2018)	N/A
Human haploid cell line, Hap1 PLEKHA5-KO	Citi Laboratory (Sluysmans et al., 2021)	N/A
Human haploid cell line, Hap1 PLEKHA5/7-KO	Citi Laboratory (Sluysmans et al., 2021)	N/A
**Recombinant DNA**		
pcDNA3.1(zeo +) human PDZD11-HA	Citi Laboratory (Guerrera et al., 2016)	S1766
pcDNA3.1(zeo +) GFP-human PDZD11-myc	Citi Laboratory (Guerrera et al., 2016)	S1744
pTRE2hyg YFP-human PLEKHA7-myc	Citi Laboratory (Paschoud et al., 2014)	S1431
pTRE2hyg YFP-human PLEKHA7-Nter (1-562)-myc	Citi Laboratory (Paschoud et al., 2014)	S1432
pTRE2hyg YFP-human PLEKHA7-ΔPH (1-162 + 282-1121)-myc	Citi Laboratory (Shah et al., 2018)	S1826
pcDNA3.1(–) GFP-myc	Citi Laboratory (Paschoud et al., 2011)	S1166
pTRE2hyg YFP-myc	Citi Laboratory (Paschoud et al., 2014)	S1210
pcDNA3.1(–) GFP-human PLEKHA5-FL	Citi Laboratory (Sluysmans et al., 2021)	S2530
pcDNA3.1(–) GFP-human PLEKHA5-WW-PH (1-270)	Citi Laboratory (This paper)	S2482
pcDNA3.1(–) GFP-human PLEKHA5-Nter (1-504)	Citi Laboratory (This paper)	S2483
pcDNA3.1(–) GFP-human PLEKHA5-Cter (410-1116)	Citi Laboratory (This paper)	S2522
pcDNA3.1(–) GFP-human PLEKHA5-PH (164-270)	Citi Laboratory (This paper)	S2565
pcDNA3.1(–) GFP-human PLEKHA5-ΔPH (1-169 + 271-1116)	Citi Laboratory (This paper)	S2523
pcDNA3.1(–) GFP-human PLEKHA5-ΔWW (107-1116)	Citi Laboratory (This paper)	S2528
pcDNA3.1(–) GFP-human PLEKHA5-FL ΔCC (1-633 + 680-1116)	Citi Laboratory (This paper)	S2654
pcDNA3.1(–) GFP-human PLEKHA5-Cter ΔCC (410-633 + 680-1116)	Citi Laboratory (This paper)	S2660
pcDNA3.1(–) GFP-human PLEKHA6-FL	Citi Laboratory (Sluysmans et al., 2021)	S2531
pcDNA3.1(–) GFP-human PLEKHA6-WW-PH (1-284)	Citi Laboratory (This paper)	S2487
pcDNA3.1(–) GFP-human PLEKHA6-Nter (1-520)	Citi Laboratory (This paper)	S2488
pcDNA3.1(–) GFP-human PLEKHA6-Cter (515-1297)	Citi Laboratory (This paper)	S2525
pcDNA3.1(–) GFP-human PLEKHA6-PH (158-284)	Citi Laboratory (This paper)	S2566
pcDNA3.1(–) GFP-human PLEKHA6-ΔPH (1-163 + 285-1297)	Citi Laboratory (This paper)	S2526
pcDNA3.1(–) GFP-human PLEKHA6-ΔWW (98-1297)	Citi Laboratory (This paper)	S2529
pcDNA3.1(–) GFP-human PLEKHA6-FL ΔCC (1-724 + 774-1297)	Citi Laboratory (This paper)	S2655
pcDNA3.1(–) GFP-human PLEKHA6-Cter ΔCC (515-724 + 774-1297)	Citi Laboratory (This paper)	S2661
pcDNA3.1(–) GFP-human PLEKHA7-FL ΔCC (1-685 + 740-1121)	Citi Laboratory (This paper)	S2657
pcDNA3.1(–) GFP-human PLEKHA7-Cter ΔCC (500-685 + 740-1121)	Citi Laboratory (This paper)	S2663
pcDNA3.1(–) GFP-human PLEKHA5-WW(1-106):PLEKHA6(98-1297) (chimera)	Citi Laboratory (This paper)	S2538
pcDNA3.1(–) GFP-human PLEKHA5-WW(1-106):PLEKHA7(97-1121) (chimera)	Citi Laboratory (This paper)	S2539
pcDNA3.1(–) GFP-human PLEKHA6-PH(164-284):PLEKHA5(1-169/271-1116) (chimera)	Citi Laboratory (This paper)	S2532
pcDNA3.1(–) GFP-human PLEKHA7-PH(165-284):PLEKHA5(1-169/271-1116) (chimera)	Citi Laboratory (This paper)	S2533
pcDNA3.1(–) GFP-human PLEKHA5-PH(170-270):PLEKHA6(1-163/285-1297) (chimera)	Citi Laboratory (This paper)	S2534
pcDNA3.1(–) GFP-human PLEKHA7-PH(165-284): PLEKHA6(1-163/285-1297) (chimera)	Citi Laboratory (This paper)	S2535
pcDNA3.1(–) GFP-human PLEKHA5-PH(170-270):PLEKHA7(1-164/285-1121) (chimera)	Citi Laboratory (This paper)	S2536
pcDNA3.1(–) GFP-human PLEKHA6-PH(164-284): PLEKHA7(1-164/285-1121) (chimera)	Citi Laboratory (This paper)	S2537
pTRE2hyg YFP-human PLEKHA7-Cter (500-1121)-myc	Citi Laboratory (This paper)	S1897
pTRE2hyg YFP-human PLEKHA7-PH (160-300)-myc	Citi Laboratory (This paper)	S1899
**Softwares and Algorithms**		
FIJI	Schindelin et al. (2012). Nature Methods 9(7): 676-682.	imagej.net/Fiji
Adobe Photoshop	N/A	adobe.com
Adobe Illustrator	N/A	adobe.com
Image Studio Lite	LI-COR	www.licor.com/bio/image-studio-lite/
SnapGene	N/A	snapgene.com/
GraphPad Prism 8	N/A	graphpad.com/scientific-software/prism/

### Cell Culture

Culture conditions for mouse cortical collecting duct cells (mCCD Tet-on) (Vasileva et al., 2017), Madin-Darby canine kidney cells (MDCKII Tet-off) (Vasileva et al., 2017) and haploid human cells (Hap1) (Popov et al., 2015) were described previously. PLEKHA6-KO, PLEKHA7-KO, PLEKHA6/7-KO, and PDZD11-KO mCCD, PLEKHA5-KO and PLEKHA6-KO MDCK, as well as PLEKHA5-KO, PLEKHA7-KO, and PLEKHA5/7-KO Hap1 were obtained by CRISPR/Cas9 genomic editing (Popov et al., 2015; Guerrera et al., 2016; Shah et al., 2018; Sluysmans et al., 2021). MDCK cysts were grown in Matrigel as described previously (Sluysmans et al., 2021).

To inhibit proteasome-mediated degradation, cells were treated with 25 μM of MG132 (Sigma-Aldrich, C2211) for 8 h at 37°C prior to lysis, using DMSO (maximal final concentration of 0.1%) as negative control.

### Antibodies

The primary antibodies targeting the following proteins, raised in the detailed host species, were used at the indicated dilution for either immunoblotting (IB) or immunofluorescence (IF): rabbit PDZD11 (Rb29958, in-house (Guerrera et al., 2016), IB: 1:1000); guinea pig PLEKHA7 (GP2737, in-house (Guerrera et al., 2016), IF: 1:500); mouse β-tubulin (32-2600, Thermo Fisher Scientific, IB: 1:3500); mouse α-tubulin (32-2500, Thermo Fisher Scientific, IF: 1:250); guinea pig α-tubulin (AA345 scFv-F2C, Geneva Antibody Facility, IF: 1/500); rabbit GFP (A-11122, Thermo Fisher Scientific, IF: 1:200); mouse HA (32-6700, Thermo Fisher Scientific, IF: 1:150); mouse E-cadherin (610181, BD Biosciences, IF: 1/2500); rat ZO-1 (R40.76, a kind gift from Prof. Daniel Goodenough (Harvard Medical School, United States), IF: 1:100).

Secondary antibodies for IF, from Jackson ImmunoResearch and diluted at 1/300, were: anti-rabbit (711-545-152) Alexa Fluor 488; anti-mouse (715-165-151) Cy3; anti-guinea pig (706-605-148) Alexa Fluor 647; anti-rat (712-175-153) Cy5. Anti-mouse and anti-rabbit (1:20000, Promega, W402B and W401B, respectively) HRP-conjugated secondary antibodies were used for IB.

### Plasmids

Constructs of full-length (FL) HA-tagged human PDZD11 (Guerrera et al., 2016), GFP-tagged human PDZD11 (Guerrera et al., 2016), human PLEKHA5 (Sluysmans et al., 2021), human PLEKHA6 (Sluysmans et al., 2021), and GST-tagged PH domain (120-300) of PLEKHA7 (Guerrera et al., 2016) have been described previously, such as GFP- and myc-tagged full-length (1-1121) (Paschoud et al., 2014), N-terminal (1-562) (Paschoud et al., 2014) and ΔPH (1-162 + 282-1121) (Shah et al., 2018) constructs of human PLEKHA7. GFP-tagged truncated and mutant constructs of PLEKHA5 (WW-PH: 1-270; Nter: 1-504; Cter: 410-1116; ΔPH: 1-169 + 271-1116; PH: 164-270; ΔWW: 107-1116; FL ΔCC: 1-633 + 680-1116; Cter ΔCC: 410-633 + 680-1116), PLEKHA6 (WW-PH: 1-284; Nter: 1-520; Cter: 515-1297; PH: 158-284; ΔWW: 98-1297; ΔPH: 1-163 + 285-1297; FL ΔCC: 1-724 + 774-1297; Cter ΔCC: 515-724 + 774-1297) and PLEKHA7 (FL ΔCC: 1-685 + 740-1121; Cter ΔCC: 500-685 + 740-1121) were obtained by PCR (triple PCR for ΔPH and ΔCC) and subcloning into pcDNA3.1(–) plasmid (*Not*I-*Kpn*I for PLEKHA5, *Not*I-*Hin*dIII for PLEKHA6 and –7) previously modified to contain N-terminal GFP (pcDNA3.1-GFP) (Paschoud et al., 2011). Mutants of PLEKHA7 (Cter: 500-1121; PH: 160-300), tagged with GFP in N-terminal and myc at the C-terminus, were made by PCR and subcloning into *Xma*I-*Xho*I (Cter) or *Not*I-*Cla*I (PH) site of a pTre2Hyg plasmid already containing GFP-myc (Paschoud et al., 2014). GFP-tagged chimeric constructs fusing the WW domains of PLEKHA5 (1-106) to PLEKHA6 (98-1297) or PLEKHA7 (97-1121) were generated by triple PCR before insertion in the *Not*I-*Hin*dIII site of pcDNA3.1-GFP (Paschoud et al., 2011). GFP-marked chimeras switching the PH domain of PLEKHA5 (170-270), PLEKHA6 (164-284) and PLEKHA7 (165-284) in the background of the others (PLEKHA5: 1-169/271-1116; PLEKHA6: 1-163/285-1297; PLEKHA7: 1-164/285-1121) were obtained by fusing PCR-amplified fragments, before subcloning into pcDNA3.1-GFP (Paschoud et al., 2011) (*Not*I-*Kpn*I/*Hin*dIII). GST-tagged PH domains of PLEKHA5 (120-270) and PLEKHA6 (120-285) were cloned into pGEX4T1 (*Eco*RI/*Bam*HI-*Not*I for PLEKHA5; *Eco*RI-*Not*I for PLEKHA6) for IPTG-inducible bacterial expression. All constructs were validated by sequencing (Microsynth, Switzerland).

### Cell Transfection and Immunofluorescence

For immunofluorescence (IF) staining, cells were seeded either on 6.5 mm/0.4 μm pore polycarbonate 24-well tissue culture inserts (Transwell filters; Corning Costar #3470), or on 12-mm glass coverslips in 24-well plates. For Hap1 cells, coverslips were coated with 0.01% Poly-L-lysine (Sigma-Aldrich, P4707) for 30 min at 37°C prior to plating. To study the localization of proteins exogenously expressed, cells at 60 to 80% confluence were transfected 1 day after seeding, using jetOPTIMUS (Polyplus) following the manufacturer’s guidelines. The day of IF staining, 48–72 h later in case of transfection, cells on coverslips were washed twice with room temperature (RT), or pre-heated at 37°C when observing microtubule staining, phosphate buffered saline (PBS) before methanol fixation during 8 min at −20°C. After three PBS washes, cells were permeabilized 5 min in PBS/Triton X-100 (0.3%) and blocked 20 min in blocking buffer [PBS/Gelatin 0.2%/Bovine serum albumin (BSA) 1%/Triton X-100 (0.03%)] prior to incubation with primary antibodies, diluted in blocking buffer, during 16 h at 4°C. Following three washes with PBS/Triton X-100 (0.3%) and 15 min of blocking, secondary antibodies and DAPI (1 μg/ml), diluted in blocking buffer, were applied during 30 min at 37°C, before final washes with PBS/Triton X-100 (0.3%) (three times) and PBS, and mounting with Fluoromount-G (Invitrogen).

Cells grown on transwells were fixed by an overnight incubation in methanol at −20°C, followed by a 1-min treatment with acetone pre-cooled at −20°C, and then stained as described previously (Sluysmans et al., 2021). Cysts were fixed with methanol and acetone mixed 1:1 for 11 min at −20°C before permeabilization with PBS containing 0.5% Triton X-100 (10 min at RT). Immunostaining was then performed as described previously (Spadaro et al., 2017). Four immunostainings were performed, from two independent cultures of cysts. Colocalization between PDZD11 and E-cadherin or α-tubulin was quantified by Pearson’s correlation coefficients. Coefficients were determined using the *Colocalization Threshold* plug-in in FIJI software, applying auto-thresholding from the Costes method (images with “Pearson’s below threshold” superior to 0.1 were not considered). Individual cysts were used to calculate the statistics.

Slides were imaged on a Zeiss LSM800 confocal microscope using a 63×/1.4NA oil immersion objective. Staining of nuclei with DAPI is colored in blue. Unless otherwise stated, scale bar = 20 μm.

### Cell Lysates and Immunoblot Analysis

Cell lysates were obtained in 500μl of RIPA buffer (NaCl 150 mM/Tris-HCl 40 mM, pH 7.5/EDTA 2 mM/glycerol 10%/Triton X-100 1%/sodium deoxycholate 0.5%/SDS 0.2%) supplemented with protease inhibitor cocktail (Thermo Fisher Scientific, A32965) from 10-cm dishes, followed by sonication (8 s at 66% amplitude with a Branson sonifier). Solubilized proteins were clarified by centrifugation (15 min at 4°C, 13 000 rpm).

Samples were mixed with SDS loading buffer and boiled 5 min at 95°C before SDS-PAGE separation at 4°C. Proteins were transferred onto nitrocellulose (0.45 μm) membrane (100 V for 80 min or 70 V for 180 min, at 4°C), and blots were blocked in Tris Buffered Saline (TBS)/Tween-20 0.1%/Low-fat milk 20% before incubation with primary antibody (diluted in TBS/Tween-20 0.1%/Low-fat milk 10%) followed by secondary HRP-labeled antibody (same buffer), and finally chemiluminescence (ECL) revelation which was detected using Odyssey Imager (LI-COR). Numbers on the left of immunoblots correspond to sizes in kDa. To compare PDZD11 expression between WT and KO cells treated with DMSO (control) or MG132, quantification of protein levels was carried out in Image Studio Lite (LI-COR), using β-tubulin signal for normalization.

### Protein-Lipid Overlay Assay

Glutathione S-transferase (GST, control) and GST-tagged PH domain of PLEKHA5, PLEKHA6 and PLEKHA7 were produced in *E. coli* (BL21-DE3): bacteria were transformed by heat shock with pGEX4T1 constructs and expression was induced with 0.1 mM IPTG for 2–5 h at 30°C. Bacterial pellets were snap frozen in liquid nitrogen before lysis in lysis buffer (PBS/Triton X-100 1%) supplemented with protease inhibitor cocktail (Thermo Fisher Scientific, A32965) and sonication five times at 55% (Branson sonifier). Cell debris were removed by centrifugation (13 000 rpm) during 15 min at 4°C. Recombinant proteins were purified by binding with Pierce Glutathione Magnetic Agarose Beads (Thermo Fisher Scientific, #78602), previously washed twice with equilibration buffer (Tris-HCl 125 mM, pH 7.4/NaCl 150 mM/DTT 1 mM/EDTA 1 mM), for 1 h 30 min at RT. After incubation, beads were washed three times with PBS before four consequent elutions (50, 40, 30 and 20 min, respectively) at RT with elution buffer (Tris-HCl 50 mM, pH 9/Reduced glutathione 30 mM). Elution products were pooled and dialyzed overnight at 4°C into PBS using a 3.5 kDa-MWCO dialysis membrane (Spectra/Por3 3,500 MWCO 18mm, #132720). Purified recombinant PH domains were quantified by Coomassie staining of SDS-PAGE, using BSA as quantitative scale. PIP strips (Echelon Biosciences, P-60001) were blocked with TBS/Tween-20 0.1%/ovalbumin (OAB) (Sigma) 0.1% for 1 h at RT before incubation overnight at 4°C with 1 μg/ml of recombinant GST-tagged PH domain diluted in TBS/Tween-20 0.1%/OAB 0.1%. 0.5 μg/ml of GST-tagged PH domain of PLC-∂1, provided by the manufacturer, was used as positive control. Membranes were then washed six times (5 min each) with TBS/Tween-20 0.1% before probing with rabbit anti-GST (Zymed, 71-7500, 1/1000) diluted in the same OAB-containing buffer for 3 h at RT. After three washes over 30 min with TBS/Tween-20 0.1%, secondary HRP-conjugated anti-rabbit was applied for 1 h at RT before final washes with TBS/Tween-20 0.1%, ECL revelation and detection using Odyssey Imager (LI-COR).

### Statistical Analysis

Statistical significance was determined using GraphPad Prism 8 software; sample size (*n*), *p*-values (*P*) and statistical tests performed are specified in figure legends. Values are shown as mean ± SD. Significance corresponds to ^∗^*p* < 0.05, ^∗∗^*p* < *0.01*, ^∗∗∗^*p* < 0.001, and ^****^p < 0.0001. For multiple comparison, after ANOVA showing significant difference, Dunnett’s test was used to compare every mean to control mean.

## Results

### The WW Domains of PLEKHA5 Are Necessary and Sufficient for Association of WW-PLEKHAs With Cytoplasmic Microtubules

WW-PLEKHAs include PLEKHA5, PLEKHA6 and PLEKHA5, and are members of the PLEKHA family that comprise isoforms that contain WW (Trp-Trp) domains (Sluysmans et al., 2021; Figures 1A–C). PLEKHA5 is detected along the lateral plasma membrane of polarized epithelial cells but is also associated with PDZD11 along cytoplasmic microtubules (Sluysmans et al., 2021). To examine the role of the N-terminal WW domains of PLEKHA5 in this latter localization, we expressed full-length, truncated and chimeric proteins in polarized epithelial kidney cells. Full-length PLEKHA5, but not a mutant lacking the WW domains, colocalized with cytoplasmic microtubules, whereas a N-terminal fragment of PLEKHA5 comprising the WW and PH domains co-localized with microtubules in the cytoplasm (arrows and arrowheads in Figure 1D and magnification insets in Figure 1D′). PLEKHA6 localizes at AJ and along lateral contacts (Sluysmans et al., 2021). The apical junctional complex (AJC) in polarized epithelial cells comprises adherens junctions (AJ, also called *zonulae adhaerentes* in epithelia) and tight junctions (TJ). Here we use either the TJ marker ZO-1, or the AJ marker PLEKHA7, or the AJ marker E-cadherin as a marker for the AJC, or briefly, for “junctions” or “cell-cell junctions.” E-cadherin is also used as a marker for the lateral domain of the plasma membrane, below the AJC. A PLEKHA6 mutant lacking the WW domains was still detected at junctions, but also in a cytoplasmic localization, distinct from microtubules (ΔWW, arrows and arrowheads, Figures 1E,E′), suggesting that the WW domains stabilize the localization along cell-cell contacts. Instead, an isolated N-terminal fragment of PLEKHA6 comprising the WW and PH domains showed a cytoplasmic diffuse localization and was not localized either at junctions or along microtubules (WW-PH, cyt., Figures 1E,E′). PLEKHA7 is exclusively detected at cell-cell junctions (Pulimeno et al., 2010). Deletion of the WW domains of PLEKHA7 does not affect PLEKHA7 localization at junctions, and the WW domains of PLEKHA7 do not localize either at junctions or along microtubules (Rouaud et al., 2020b). In contrast, chimeric proteins of PLEKHA6 and PLEKHA7, where the endogenous WW domains were replaced by the WW domains of PLEKHA5, colocalized with cytoplasmic microtubules (arrows, Figures 1F,F′). Together, these results indicate that the WW domains of PLEKHA5 are necessary and sufficient, also in the context of a chimeric WW-PLEKHA, to drive their association with microtubules.

### The Localization of PLEKHA5 and PDZD11 Along Cytoplasmic Microtubules Is Mutually Interdependent

**FIGURE 2:**
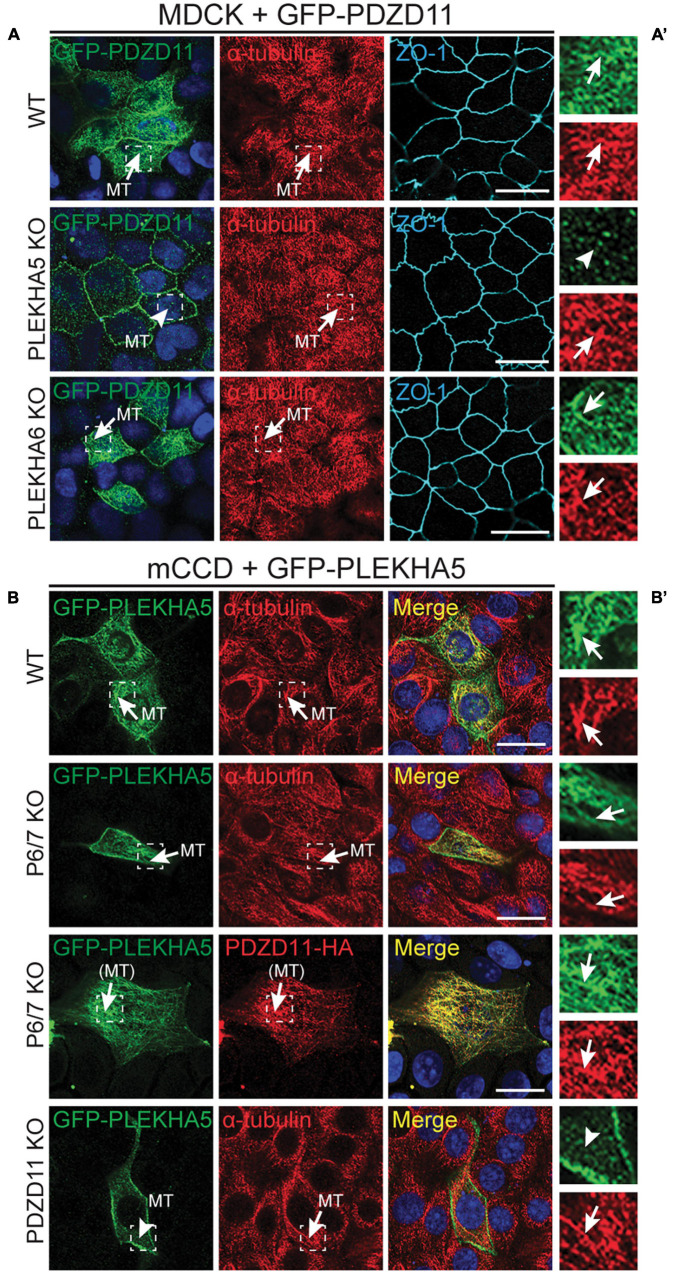
The interaction between the tandem WW domains of PLEKHA5 and PDZD11 is necessary and sufficient for their localization along cytoplasmic microtubules. **(A)** IF analysis of the localization of GFP-tagged PDZD11 in MDCK cells, either WT, or PLEKHA5 KO, or PLEKHA6 KO. α-tubulin is used as a marker for microtubules and ZO-1 as a marker for cell-cell junctions. **(B)** IF analysis of the localization of GFP-tagged PLEKHA5 full-length in mCCD WT, PLEKHA6-PLEKHA7 double KO (P6/7 KO) and PDZD11 KO. α-tubulin is used as a marker for microtubules. Squares correspond to enlarged areas of green and red channels in panels **(A′,B′)**. Microtubules (MT) and microtubules-like fibrils [(MT)] are indicated, with arrows showing labeling and arrowheads low/undetectable labeling. Scale bar = 20 μm.

Although labeling of epithelial cells with anti-PDZD11 antibodies shows accumulation at cell-cell junctions, previous results indicate that both PLEKHA5 and PDZD11 are associated with cytoplasmic microtubules (Sluysmans et al., 2021). To clarify the mechanism for the cytoplasmic localization of PDZD11, we first examined the localization of exogenous GFP-tagged PDZD11 either in WT cells, or cells KO for either PLEKHA5 or PLEKHA6. The KO of PLEKHA5, but not PLEKHA6, in MDCK cells resulted in the loss of cytoplasmic PDZD11 labeling (arrows and arrowheads, Figures 2A,A′), indicating that PLEKHA5 but not PLEKHA6 is required for the localization of PDZD11 along cytoplasmic microtubules. Next, to determine if PDZD11 controls the cytoplasmic microtubules-associated localization of PLEKHA5, we expressed GFP-tagged PLEKHA5 in WT cells or in cells KO for either PDZD11 or PLEKHA6 and PLEKHA7. In WT cells and in PLEKHA6/7-double KO cells, exogenous PLEKHA5 accumulated in fibrillar cytoplasmic filaments that colocalized with microtubules and exogenous PDZD11 (Figures 2B,B′). In contrast, GFP-PLEKHA5 was detected diffusely in the cytoplasm, and not associated with microtubules in PDZD11-KO cells (arrowhead, Figure 2B, PDZD11 KO). Together, these results indicate that the localization of PLEKHA5, and of chimeric molecules comprising the WW domains of PLEKHA5, along cytoplasmic microtubules depends on the interaction of the WW domains of PLEKHA5 with PDZD11.

### WW-PLEKHAs Stabilize Different Pools of PDZD11

We hypothesized that WW-PLEKHAs stabilize different pools of PDZD11 at different subcellular localizations (Sluysmans et al., 2021). To investigate in more details the role of WW-PLEKHAs in the regulation of PDZD11, we analyzed the localization of endogenous and exogenous PDZD11, and the protein expression levels of endogenous PDZD11 in cells KO for WW-PLEKHAs, grown either in a 3D cyst culture model, or polarized on transwell filters.

**FIGURE 3:**
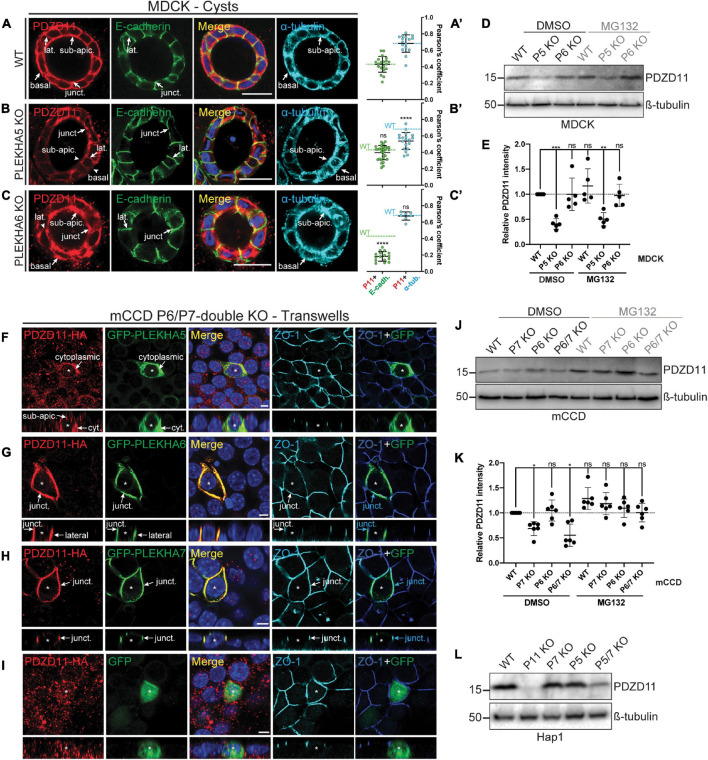
PLEKHA5, PLEKHA6, and PLEKHA7 define and stabilize cellular pools of PDZD11 with distinct localizations. **(A–C)** IF analysis of endogenous PDZD11 localization in MDCK WT **(A)**, PLEKHA5 KO **(B),** and PLEKHA6 KO **(C)** cells grown in Matrigel as 3D cysts. α-tubulin and E-cadherin show microtubular and lateral/junctional staining, respectively. Basal, junctional (junct.), lateral (lat.) and sub-apical (sub-apic.) localizations are indicated, with arrows showing labeling and arrowheads low/undetectable labeling. In **(A′–C′)**,colocalization between PDZD11 (P11) and E-cadherin (E-cadh., green) or α-tubulin (α-tub., cyan) is quantified by Pearson’s correlation coefficients. Dashed lines indicate the mean of the WT cells for P11-E-cadh. (green) or P11-α-tub. (cyan). Dots show replicates corresponding to individual cysts (*n*: E-cadh.: WT: 20, PLEKHA5 KO: 29, PLEKHA6 KO: 16; α-tub.: WT: 16, PLEKHA5 KO: 20, PLEKHA6 KO: 7), and bars represent mean ± SD. Ordinary one-way ANOVA with *post hoc* Dunnett’s test to compare to WT (ns, not significant, ****p < 0.0001). **(D,E,J–L)** IB analysis **(D,J,L)** and quantification **(E,K)** of PDZD11 protein expression in WT, PLEKHA5 (P5) KO and PLEKHA6 (P6) KO MDCK cells **(D,E)**, in WT, PLEKHA7 (P7) KO, P6 KO, and P6/P7 double KO mCCD cells **(J,K)**, and in WT, PDZD11 (P11) KO, P7 KO, P5 KO, and P5/7 double KO Hap1 cells **(L)**, upon treatment with the proteasome inhibitor MG132 (DMSO as control; ß-tubulin serves as loading normalization). In panels **(E,K)** dots show replicates (ß-tubulin-normalized PDZD11 signals relative to DMSO-treated WT cells) and bars represent mean ± SD. Repeated measures (RM) one-way ANOVA with *post hoc* Dunnett’s test (ns, not significant; *p < 0.05, **p < *0.01*, and ***p < 0.001). **(F–I)** IF analysis of the localization of exogenous HA-tagged PDZD11 (PDZD11-HA) upon co-transfection either with GFP-PLEKHA5 **(F)**, or GFP-PLEKHA6 **(G)**, or GFP-PLEKHA7 **(H)**, or GFP alone **(I)** in PLEKHA6-PLEKHA7 double KO mCCD cells grown on transwells. Bottom panels show XZ sections taken at the horizontal middle of the XY plane. Cytoplasmic (cyt.), junctional (junct.), lateral and sub-apical (sub-apic.) localizations are indicated. Asterisks show transfected cells. ZO-1 is used as a junctional marker, and merge panels between ZO-1 (in blue) and GFP (in green) show junctional localization (pointed by cyan arrows). Scale bars = 20 μm **(A–C)** or 5 μm **(F–I)**.

In WT MDCK cysts, endogenous PDZD11 was detected in the sub-apical cytoplasm, along lateral contacts, at junctions and near the basal membrane (Figure 3A). The distribution of PDZD11 was broadly similar to that of α-tubulin, whereas co-localization between E-cadherin and PDZD11 was detected along lateral contacts and at junctions (arrows, Figure 3A, quantification of the colocalization (positive Pearson’s correlation coefficients of the signals) between PDZD11 and E-cadherin or α-tubulin in Figure 3A′). The KO of PLEKHA5 resulted in a dramatic decrease of endogenous PDZD11 labeling in the cytoplasm, especially in the sub-apical and near-basal regions, whereas labeling was still detected at AJ and along lateral contacts (Figure 3B, quantification in Figure 3B′). In contrast, in cells KO for PLEKHA6 no lateral contact labeling for endogenous PDZD11 was detected, whereas PDZD11 was still localized in the cytoplasm and near apical and basal domains of the plasma membrane (Figure 3C, quantification in Figure 3C′). The distribution of microtubules, as detected by α-tubulin staining, was not significantly altered by KO of either PLEKHA5 or PLEKHA6 (Figures 3A–C). IB analysis showed that PDZD11 protein levels were decreased in PLEKHA5-KO but not PLEKHA6-KO cells, and that treatment with the proteasome inhibitor MG132 did not rescue PDZD11 protein levels (Figure 3D, quantification in Figure 3E).

Next, we analyzed the role of WW-PLEKHAs in the localization of PDZD11 using mCCD cells KO for both PLEKHA6 and PLEKHA7, asking how exogenous expression of WW-PLEKHAs affected the localization of exogenous PDZD11. Expression of exogenous PLEKHA5 enhanced the accumulation of exogenous PDZD11 in the cytoplasm (arrows, Figure 3F). Expression of PLEKHA6 enhanced the accumulation of PDZD11 along junctional and lateral plasma membranes (arrows, Figure 3G). Expression of PLEKHA7 enhanced PDZD11 accumulation at cell-cell junctions (arrows, Figure 3H), whereas expression of GFP alone did not result in accumulation of exogenous PDZD11-HA at any specific localization (Figure 3I). Moreover, IB analysis showed that in mCCD cells KO for either PLEKHA7, or both PLEKHA6 and PLEKHA7, there was a decrease in PDZD11 protein levels which was rescued by the proteasome inhibitor MG132 (Figure 3J, quantification in Figure 3K). IB analysis also showed that in Hap1 cells, double-KO for PLEKHA5 and PLEKHA7 resulted in significantly decreased protein levels of PDZD11 (Figure 3L). Collectively, these results suggest that PLEKHA5 and PLEKHA7 redundantly stabilize cytoplasmic and junction-associated pools of PDZD11, respectively, and that PLEKHA7 prevents the proteasomal degradation of PDZD11.

### The PH Domains of WW-PLEKHAs Promote Their Localization at Lateral and Junctional Membranes

**FIGURE 4:**
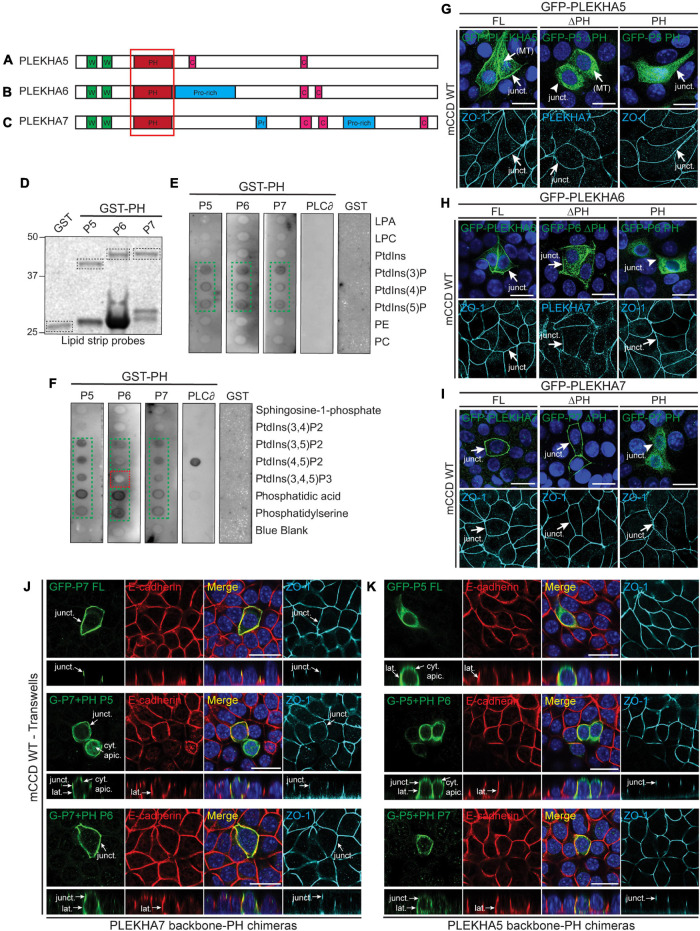
Phospholipid binding and cellular localizations of the PH domains of WW-PLEKHAs. **(A–C)** Scheme of the structural organization of PLEKHA5 **(A)**, PLEKHA6 **(B)**, and PLEKHA7 **(C)** showing the domains: WW (Trp-Trp) (W) in green, PH (pleckstrin homology) in red, Proline-rich (Pro-rich or Pr) in blue, coiled-coil **(C)** in pink. Red box indicates the structural domain analyzed. **(D–F)** *In vitro* interaction of PH domains of WW-PLEKHAs with phospholipids. Coomassie staining **(D)** of purified GST fusions of PH domains of PLEKHA5 (P5), PLEKHA6 (P6) and PLEKHA7 (P7) (framed bands), and **(E,F)** IB analysis of lipid-protein overlay assay (GST-PH domain of phospholipase C-∂1 (PLC∂) as positive control, GST alone as negative control). Green frames highlight binding, red frames indicate low/undetectable interaction. LPA, lysophosphatidic acid; LPC, lysophosphocholine; PtdIns, phosphatidylinositol; P, phosphate; P2, biphosphate; P3, triphosphate; PE, phosphatidylethanolamine; PC, phosphatidylcholine. **(G–I)** IF analysis of the localization of exogenous GFP-tagged constructs of PLEKHA5 **(G)**, PLEKHA6 **(H)**, and PLEKHA7 **(I)** in WT mCCD cells. Either full-length (FL), or FL with a deletion of the PH domain (ΔPH), or PH domain alone (PH) were used. ZO-1 and PLEKHA7 are used as junctional markers. Junctional (junct.) and fibrillar microtubules-like [(MT)] localizations are indicated. Arrows show labeling, arrowheads indicate low/undetectable labeling. **(J,K)** IF analysis of the localization of either GFP-tagged PLEKHA7 (P7) **(J)** or PLEKHA5 (P5) **(K)** constructs in WT mCCD cells. Either full-length (FL) proteins, or chimeras where the PH domain was replaced [either from P5 or P6 in PLEKHA7 **(J)**, or from P6 or P7 for PLEKHA5 **(K)**] are shown. mCCD cells were polarized on transwells and XZ section were taken at the horizontal middle of the XY plane (square panels). Cytoplasmic sub-apical (cyt. apic.), junctional (junct.), and lateral (lat.) localizations are indicated. E-cadherin and ZO-1 are used as lateral and junctional markers, respectively. Scale bar = 20 μm.

Pleckstrin homology (PH) domains have been implicated in targeting proteins to membranes, through the recognition of specific phospholipid membrane components (Lemmon, 2008). To analyze the role of the PH domains of WW-PLEKHAs (Figures 4A–C) in their localization, we generated construct for the bacterial expression of GST fused to the PH domain of each of the WW-PLEKHAs (Figure 4D) and analyzed their lipid-binding properties using PIP strips. Strong interactions were observed between the GST fusions of the PH domains of PLEKHA5, PLEKHA6 and PLEKHA7 with phosphatidylinositol (3)-phosphate PtdIns(3)P, PtdIns(4)P, PtdIns(5)P, PtdIns(3,5)P2, PtdIns(4,5)P2, phosphatidic acid and phosphatidylserine (Figures 4E,F). However, only PLEKHA5 and PLEKHA7, but not PLEKHA6, bound to PtdIns(3,4,5)P3 (Figure 4F). PLEKHA5 weakly interacted with PtdIns(3,4)P2 (Figure 4F).

Next, we examined the role of PH domains in the localization of exogenous GFP-tagged WW-PLEKHA constructs in WT mCCD cells. Deletion of the PH domain of PLEKHA5 resulted in the loss of its localization at cell-cell contacts (arrowhead, ΔPH, Figure 4G), whereas its cytoplasmic microtubules-associated localization was not affected by the deletion of the PH domain. The isolated PH domain of PLEKHA5 was mostly distributed in the cytoplasm but also detectable, albeit weakly, at cell-cell contacts (arrow, PH, Figure 4G). In contrast, deletion of the PH domain in either PLEKHA6 or PLEKHA7 did not affect their junctional localization, and constructs containing only the PH domain of either PLEKHA6 or PLEKHA7 were not detectable at either AJs or lateral contacts (arrows and arrowheads, Figures 4H,I). This indicates that only the PH domain of PLEKHA5 is necessary and sufficient for association with the lateral plasma membrane.

To further analyze the role of PH domains, we generated chimeric molecules where heterologous PH domains were inserted in the backbone of either PLEKHA7 (Figure 4J) or PLEKHA5 (Figure 4K) or PLEKHA6 (Supplementary Figure 1). The PH domain of PLEKHA5 induced the ectopic localization of PLEKHA7 to lateral contacts and to the apical submembrane cytoplasm, whereas the PH domain of PLEKHA6 induced the ectopic localization of PLEKHA7 to lateral contacts (Figure 4J). Conversely, the PH domain of either PLEKHA6 or PLEKHA7 promoted the ectopic accumulation of PLEKHA5 at AJ (Figure 4K). Finally, chimeric PLEKHA6 molecules containing the PH domain of either PLEKHA5 or PLEKHA7 were still localized at AJ and along lateral contacts, but chimeric molecules containing the PH domain of PLEKHA5 were also detectable in the subapical cytoplasm (Supplementary Figure 1). Together, these results suggest that in cultured polarized epithelial cells the PH domain of PLEKHA5 directs WW-PLEKHAs to lateral contacts and subapical cytoplasm, and the PH domains of PLEKHA6 and PLEKHA7 promote the accumulation of chimeric proteins along lateral membranes and junctions, although they are not required for the localizations of the non-chimeric proteins at these sites.

### C-Terminal Sequences of PLEKHA6 and PLEKHA7 and the Coiled-Coil Domain of PLEKHA7 Promote Their Localization at Adherens Junctions

**FIGURE 5:**
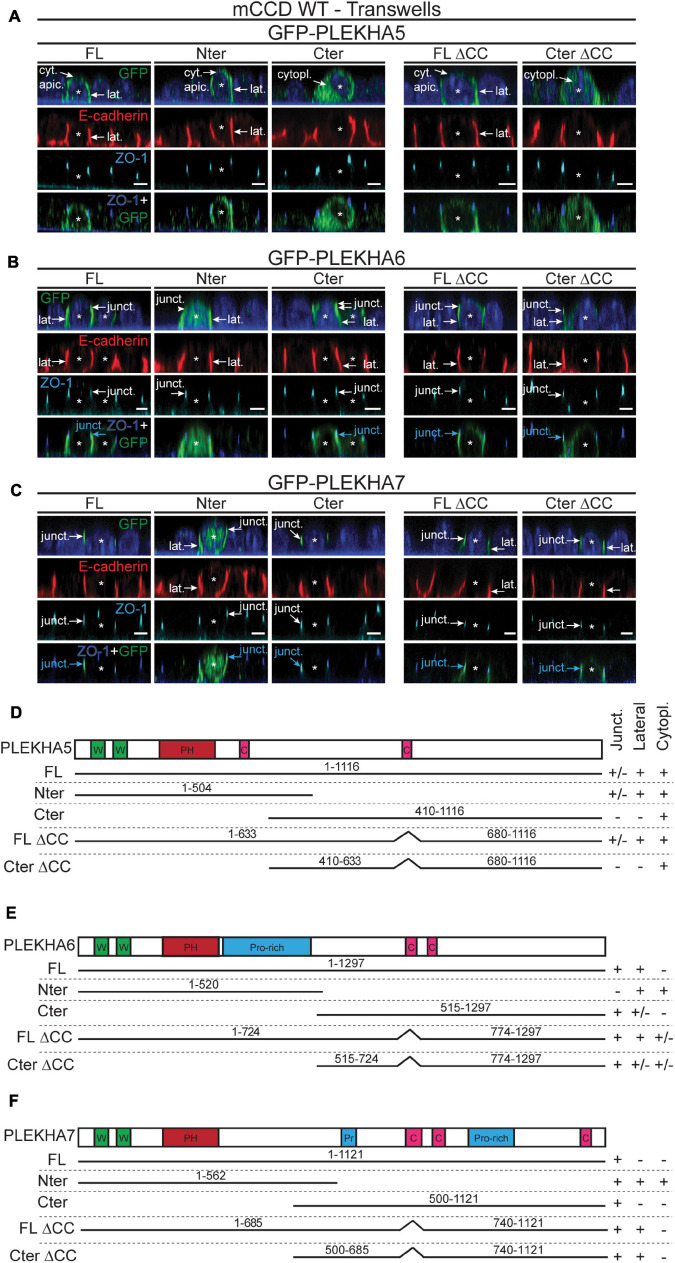
The N-terminal half of PLEKHA5 and the C-terminal halves of PLEKHA6 and PLEKHA7, respectively, are the major determinants of their distinct subcellular localizations. **(A–C)** IF analysis of the localization of GFP-tagged exogenous PLEKHA5 **(A)**, PLEKHA6 **(B)**, and PLEKHA7 **(C)** after transfection in WT mCCD cells grown on transwells. Either full-length (FL), N-terminal fragment (Nter), C-terminal fragment (Cter), or mutants of FL and Cter fragments lacking the coiled-coil region (ΔCC) were used. Asterisks show transfected cells. Cytoplasmic sub-apical (cyt. apic.), cytoplasmic (cytopl.), junctional (junct.) and lateral (lat.) localizations are indicated, with arrows showing labeling and arrowheads low/undetectable labeling. E-cadherin and ZO-1 are used as lateral and junctional markers, respectively, and merge panels between ZO-1 (in blue) and GFP (in green) show junctional localization (pointed by cyan arrows). Scale bar = 5 μm. **(D–F)** Schemes of the domain organization of PLEKHA5 **(D)**, PLEKHA6 **(E)**, and PLEKHA7 **(F)** and details (amino acid numbers are indicated) of the constructs used for transfection (N-terminal GFP is not shown for clarity), and of their localization (Junct. = junctional, Lateral, Cytopl. = cytoplasmic). W, WW domain (Trp-Trp); PH, Pleckstin Homology domain; C, coiled-coil region; Pr/Pro-rich, proline-rich region.

The C-terminal regions of WW-PLEKHAs contain coiled-coil domains (Sluysmans et al., 2021). To investigate the role of coiled-coil domains in the subcellular targeting of WW-PLEKHAs, we examined the behaviors of exogenously expressed, GFP-tagged full-length (FL) proteins, N-terminal (Nter) fragment, and C-terminal (Cter) fragment, either WT, or with a deletion of the coiled-coil domain present in the Cter fragment (IF in Figures 5A–C, schemes of constructs in Figures 5D–F). The Nter region of PLEKHA5 showed a localization indistinguishable from that of the full-length protein, whereas its Cter region was exclusively cytoplasmic, confirming that the molecular determinants of the lateral plasma membrane localization of PLEKHA5 lie within the Nter region (FL, Nter, Cter, Figures 5A,D). Deletion of the coiled-coil region within the Cter of PLEKHA5 did not affect the localization of either the full-length protein or the Cter fragment (FL ΔCC, Cter ΔCC, Figures 5A,D). In the case of PLEKHA6, the Nter region was localized both along the lateral membrane and in the cytoplasm, but not at junctions (arrows and arrowheads, Figure 5B), indicating that sequences in the Cter region are critical for junctional localization. Labeling for the Cter of PLEKHA6 was strong at junctions and weak along lateral membranes (Figures 5B,E). However, mutant FL and Cter constructs of PLEKHA6 lacking the coiled-coil region still displayed both junctional and lateral localizations (FL, Nter and Cter, and FL ΔCC, Cter ΔCC, Figures 5B,E), indicating that coiled-coil sequences are not uniquely required for either localizations. The exogenously expressed Nter region of PLEKHA7 was detected along lateral contacts in addition to junctions, while the Cter half of PLEKHA7 was exclusively localized at junctions (arrows and arrowheads, Figure 5C). Deletion of the coiled-coil domains of PLEKHA7 within the full-length protein or within the Cter region resulted in the appearance of lateral labeling, in addition to junctional labeling (arrows, FL ΔCC, Cter ΔCC, Figures 5C,F). In summary, these data support an important role for C-terminal sequences of PLEKHA6 and PLEKHA7 and for the coiled-coil region of PLEKHA7 in their accumulation at junctions.

## Discussion

WW-PLEKHAs are multidomain scaffolding proteins that have been implicated in neural and cardiac development and in various systemic and organ diseases, including neurological disorders, hypertension and cancer (Levy et al., 2009; Wythe et al., 2011; Vithana et al., 2012; Yamada et al., 2012; Fromer et al., 2014; Jamain et al., 2014; Spellmann et al., 2014; Jilaveanu et al., 2015; Kourtidis et al., 2015; Thapa et al., 2015; Tille et al., 2015; Barbitoff et al., 2018; Cox et al., 2018; Tavano et al., 2018; Huang et al., 2020; Liu et al., 2020; Nair-Menon et al., 2020). At the cellular level, PLEKHA7 has been involved in the stabilization of cadherin-based junctions and TJ barrier function through the anchorage to microtubules (Meng et al., 2008; Paschoud et al., 2014), the junctional recruitment of RNA-interference machinery and the RNA-inducing silencing complex (RISC) to regulate signaling and cancer growth (Kourtidis et al., 2015, 2017; Nair-Menon et al., 2020), and the junctional recruitment of nectins and the Tspan-ADAM10 complex of transmembrane proteins (Guerrera et al., 2016; Shah et al., 2018). All three WW-PLEKHAs (PLEKHA5, PLEKHA6, PLEKHA7) control the anterograde trafficking and localization at the cell periphery of the copper pump ATP7A in elevated copper (Sluysmans et al., 2021). WW-PLEKHAs form a complex with PDZD11 to control the localization of transmembrane proteins, and the N-terminal WW domains are critically important for this function.

The molecular mechanisms through which WW-PLEKHAs control these physiological and pathological processes are in large part not understood. However, we showed that the junctional versus lateral localization of the Tspan33-ADAM10 complex controls the cellular response to α-toxin from *Staphylococcus aureus*, and copper efflux requires the anterograde traffic of ATP7A-containing vesicles to the cell periphery (Lutsenko et al., 2007). These results highlight the concept that proper localization of transmembrane proteins at specific domains and subdomains of the plasma membrane is crucially important for their function. Thus, understanding how WW-PLEKHAs are localized in different subcellular compartments is critical to investigate the molecular basis of their function. Here we analyzed the role of WW, PH and coiled-coil domains in the localizations of PLEKHA5, PLEKHA6 and PLEKHA7 and their functional interaction with PDZD11.

The WW domains of all WW-PLEKHAs bind to the N-terminal region of PDZD11, which contains two poly-proline stretches (Rouaud et al., 2020b; Sluysmans et al., 2021). Here we confirm that the WW domains are not required for the junctional localization of either PLEKHA7 or PLEKHA6, and we show that the WW domains are required for the association of PLEKHA5 with PDZD11 along cytoplasmic microtubules. Indeed, the localization of both endogenous PLEKHA5 and PDZD11 at cytoplasmic microtubules is mutually interdependent, suggesting that the formation of the PLEKHA5-PDZD11 complex results in a conformational change, either in the WW domains of PLEKHA5, or in PDZD11, or both, that leads either to the interaction with microtubules. There is evidence that the WW domains of the isomerase Pin11 bind to the microtubule-associated protein tau (Lu et al., 1999), and that PDZ domains can also bind to microtubules (Gorovoy et al., 2005). Thus, future studies should address the basis for the direct or indirect association of the PLEKHA5-PDZD11 complex to microtubules or microtubule-associated proteins. PLEKHA7 was previously shown to tether microtubules to the adherens junctions through the minus-end microtubule binding proteins CAMSAP3/nezha (Meng et al., 2008). CAMSAP3 accumulates in the apical submembrane region in polarized epithelial cells, to direct their polarization (Toya et al., 2016). Since PLEKHA5 accumulates sub-apically in polarized epithelial cells grown in cysts (Sluysmans et al., 2021), it will be interesting to determine the role of CAMSAP3/nezha in the subcellular distribution of PLEKHA5. Functionally, the association of PLEKHA5-PDZD11 with microtubules may be relevant for a function in microtubule-dependent trafficking of transmembrane protein complexes, such as the ATP7A-WW-PLEKHA-PDZD11 complexes from the trans-Golgi network to the cell periphery.

The PH domain is a protein module of approximately 100 amino acids, which is present in a variety of proteins involved in phosphoinositide metabolism, signaling, and cytoskeletal organization (Maffucci and Falasca, 2001; Lemmon et al., 2002; Lindmo and Stenmark, 2006; Lemmon, 2007). PH domains can target proteins to the membrane, and the PH domain of Hadp1, the zebrafish homolog of PLEKHA7, has been implicated in membrane targeting of PLEKHA7 in cardiac myocytes (Wythe et al., 2011). Furthermore, PLEKHA4 localizes to the plasma membrane via recognition of PtdIns(4,5)P2 (Shami Shah et al., 2019). Our result confirm the lipid binding specificities that were previously reported for PLEKHA5 and PLEKHA7 (Dowler et al., 2000; Wythe et al., 2011; Zou and Cox, 2013), and show that PLEKHA6 has a pattern of phospholipid binding similar to that of both PLEKHA5 and PLEKHA7, except for the lack of interaction of PLEKHA6 with PtdIns(3,4,5)P3. However, the localization of WW-PLEKHA mutants lacking the PH domain and of isolated PH domains indicate that only the PH domain of PLEKHA5 is necessary and sufficient for membrane association in epithelial cells. We previously showed that different N-terminal and C-terminal fragments of PLEKHA7 are targeted to cell-cell junctions and lateral membranes (Paschoud et al., 2014), suggesting that WW and PH domains are not strictly required for junctional localization. However, the notion that the PH domain of PLEKHA7 plays a role in membrane attachment is supported by the observation that when it is present in the context of a chimeric PLEKHA5 molecule, it drives an ectopic junctional localization of the chimeric protein to cell-cell junctions. Together, these observations suggest that only the PH domain of PLEKHA5 binds to plasma membrane phosphoinositides with sufficiently high affinity and specificity to drive, by itself, membrane attachment. On the other hand, in the case of PLEKHA6 and PLEKHA7, membrane targeting requires cooperative interactions mediated not only by the PH, but also by additional domains, such as WW, proline-rich and coiled-coil domains. The *in vivo* specificity of binding of the PH domains of WW-PLEKHAs remains to be established and will be important to understand the molecular basis of the binding of WW-PLEKHAs to membranes. For example, several cytoskeletal proteins with PH domains are recruited to membrane sites that contain products generated by the activation of PI3-kinase (Lemmon et al., 2002), and formation of E-cadherin based junctions leads to the activation of PI3-kinase (Pece et al., 1999; Kovacs et al., 2002). Since PLEKHA7 is specifically associated with E-cadherin-associated junctions, PLEKHA7 may be recruited to cadherin-based junctions through cooperative interactions of the PH domain with PI3-kinase products, coupled to interactions of N-terminal and C-terminal sequences with specific junctional proteins, such as afadin (Kurita et al., 2013) and paracingulin (Pulimeno et al., 2011). Another potential role of the PH domain of WW-PLEKHAs could be the regulation of membrane traffic. PH-mediated interaction of proteins with membranes have been involved in endocytosis, recycling, micropinocytosis, and trafficking between trans-Golgi network and plasma membrane (Lindmo and Stenmark, 2006). Of note, the PH domains of the three WW-PLEKHAs interact *in vitro* with PtdIns(3)P, which has been involved in endosome traffic (Schink et al., 2013). This could be relevant for the regulation of the membrane recycling of vesicles containing the Menkes copper ATPase ATP7A. Interestingly, in polarized epithelial cells, asymmetries in the distribution of phosphoinositides have been involved in the specification of apical versus basolateral membrane identity, for example PtdIns(4,5)P2 are apical and PtdIns(3,4,5)P3 basolateral in MDCK cells (Shewan et al., 2011). In this respect, it was intriguing to observe that PLEKHA6, the only WW-PLEKHA whose PH domain does not bind to PtdIns(3,4,5)P3 *in vitro*, is present at the lateral membrane in cultured cells, whereas chimeric molecule with the backbone of PLEKHA6 and the PH domain of PLEKHA5 was targeted to the sub-apical cytoplasm. Furthermore, the chimeric protein with the PLEKHA5 backbone and the PH domain of either PLEKHA6 or PLEKHA7 was still detected sub-apically. Finally, a basal localization of endogenous PLEKHA6 was observed both in kidney and intestinal epithelial cells, but not in cultured kidney epithelial cells (Sluysmans et al., 2021). Together, these observations indicate that the sub-apical localization of PLEKHA5 and the lateral and basal localizations of PLEKHA6 depend on cooperative interactions, and not only on their PH domain phosphoinositide-binding specificities. Future studies should also investigate whether PH domains of PLEKHA proteins help establish or stabilize areas of specific lipid composition within the membrane.

Our observations indicate that sequences in the C-terminal region of PLEKHA6 and PLEKHA7 and the coiled-coil region of PLEKHA7 promote junctional targeting, whereas the isolated C-terminal region of PLEKHA5 failed to localize at either AJ or lateral contacts. In PLEKHA4, coiled-coil and intrinsically disordered regions are responsible for oligomerization and formation of liquid-liquid phase separated clusters (Shami Shah et al., 2019). Whether WW-PLEKHAs can dimerize or oligomerize through their coiled-coil domains or interact with proteins that direct their localization at junctions remains to be investigated by future studies. The possibility that WW-PLEKHAs could also form liquid-liquid phase separated clusters is suggested by bioinformatic analysis (Rouaud et al., 2020a).

PDZD11 binds to all three WW-PLEKHAs, and our data indicate that in epithelial cells PDZD11 is stabilized non-redundantly in the cytoplasm by PLEKHA5 and at junctions by PLEKHA7. The degradation of PDZD11 in PLEKHA7-KO cells can be rescued by the proteasome inhibitor MG132, suggesting that PDZD11 can be degraded by ubiquitination. In agreement, PDZD11 contains two potential ubiquitination sites in its PDZ domain. Although the mechanism through which WW-PLEKHAs protect PDZD11 from proteasomal degradation is not clear, PLEKHA4 was found to negatively regulate the E3 ubiquitin ligase CUL3 by binding to its adaptor KLHL12, resulting in increased levels of DVL and positive regulation of Wnt signaling (Shami Shah et al., 2019). Thus, future studies should investigate whether WW-PLEKHAs bind to or regulate ubiquitin ligases and whether mutation of potential ubiquitination sites protects PDZD11 from proteolytic degradation in WW-PLEKHA-KO cells.

In summary, we identified the structural regions of WW-PLEKHA proteins that direct their distinct subcellular localizations, providing a rational basis for future investigations about the machinery involved in the trafficking of transmembrane proteins by PDZD11-WW-PLEKHA complexes.

## Data Availability Statement

The original contributions presented in the study are included in the article/Supplementary Material, further inquiries can be directed to the corresponding author.

## Author Contributions

SC initiated and supervised the project. SS conducted most of the experiments. IM conducted IB, IF, and IHC experiments. IM and LJ constructed plasmids. SS and SC analyzed the data and wrote the manuscript. All authors contributed to the article and approved the submitted version.

## Conflict of Interest

The authors declare that the research was conducted in the absence of any commercial or financial relationships that could be construed as a potential conflict of interest.

## Publisher’s Note

All claims expressed in this article are solely those of the authors and do not necessarily represent those of their affiliated organizations, or those of the publisher, the editors and the reviewers. Any product that may be evaluated in this article, or claim that may be made by its manufacturer, is not guaranteed or endorsed by the publisher.

## Supplementary Material

Supplementary Figure 1 The role of PH domain in the cellular localization of PLEKHA6. IF analysis of the localization of GFP-tagged PLEKHA6 (P6) constructs in WT mCCD cells. Either full-length (FL) protein, or chimeras where the PH domain was replaced with the PH domain of either PLEKHA5 (PH P5) or PLEKHA7 (PH P7) are shown. mCCD cells were polarized on transwells and XZ section were taken at the horizontal middle of the XY plane (square panels). Cytoplasmic sub-apical (cyt. apic.), junctional (junct.) and lateral (lat.) localizations are indicated. E-cadherin and ZO-1 are used as lateral and junctional markers, respectively. Scale bar = 20 μm.
