# Supplementary figures and images for: WW, PH and C-Terminal Domains Cooperate to Direct the Subcellular Localizations of PLEKHA5, PLEKHA6 and PLEKHA7
Source: Front Cell Dev Biol. 2021 Sep 9;9:729444. doi: 10.3389/fcell.2021.729444 (PMC8458771; doi:10.3389/fcell.2021.729444)

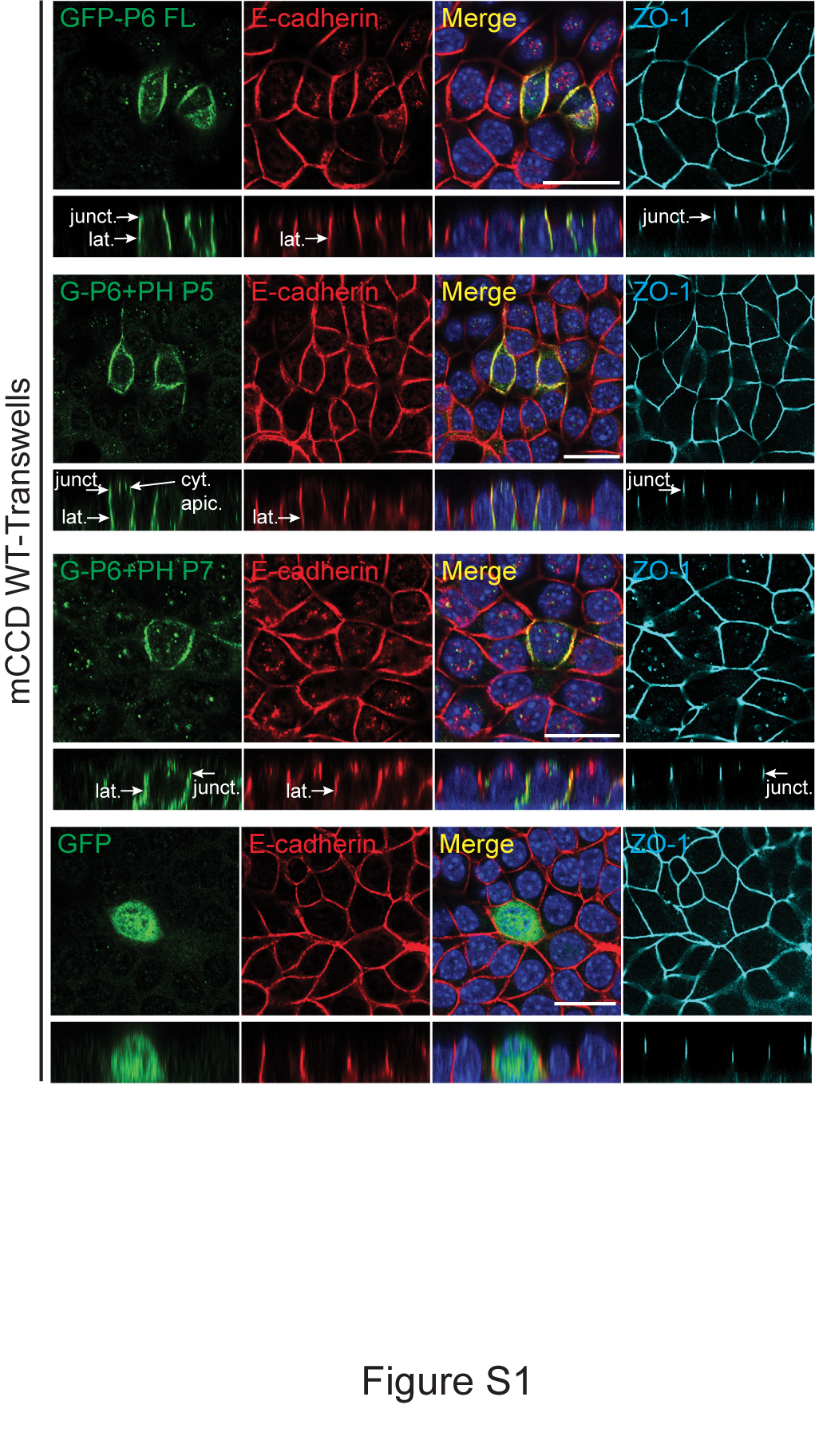

Supplement: Supplementary Figure 1 — The role of PH domain in the cellular localization of PLEKHA6. IF analysis of the localization of GFP-tagged PLEKHA6 (P6) constructs in WT mCCD cells. Either full-length (FL) protein, or chimeras where the PH domain was replaced with the PH domain of either PLEKHA5 (PH P5) or PLEKHA7 (PH P7) are shown. mCCD cells were polarized on transwells and XZ section were taken at the horizontal middle of the XY plane (square panels). Cytoplasmic sub-apical (cyt. apic.), junctional (junct.) and lateral (lat.) localizations are indicated. E-cadherin and ZO-1 are used as lateral and junctional markers, respectively. Scale bar = 20 μm. [file Image_1.TIF]
